# Disentangling the Drivers of Obesity: An Analytical Framework Based on Socioeconomic and Intrapersonal Factors

**DOI:** 10.3389/fnut.2021.585318

**Published:** 2021-03-03

**Authors:** Wisdom Dogbe, Melania Salazar-Ordóñez, Jose M. Gil

**Affiliations:** ^1^Rowett Institute, School of Medicine, Medical Sciences and Nutrition, University of Aberdeen, Aberdeen, United Kingdom; ^2^Universidad de Córdoba, WEARE-Water, Environmental and Agricultural Resources Economics, Córdoba, Spain; ^3^Center for Agro-Food Economics and Development (CREDA-UPC-IRTA), University Polytechnic of Catalonia, Barcelona, Spain

**Keywords:** body mass index, economic and sociodemographic features, attitude toward obesity, beliefs toward obesity, risk and loss aversion, non-linear robust path analysis

## Abstract

Obesity is increasing at exponential rates in developed economies despite the numerous policy interventions being implemented. The causes of obesity are multifactorial demanding a holistic review for targeted intervention. This study, therefore, provides a holistic overview of multiple factors affecting body weights i.e., socioeconomic and intrapersonal factors. We used data from a household and experimental survey carried out in Spain (Barcelona) in 2014. A non-linear path analysis was used considering the non-linear relationships that might exist between these factors and body weight. Results confirm non-linear relationships between some socioeconomic, intrapersonal factors and body weight. Among the intrapersonal factors, obesity is directly influenced by volitional control of obesity, attitude toward obese persons, holding a correct body image and body image dissatisfaction. Socioeconomic factors that have significant influence on obesity were age, education and gender. Risk attitudes do not correlate with obesity.

## Introduction

Obesity, which can be defined as an unhealthy excess of body fat (1) and measured by the Body Mass Index (BMI)^1^ (2, 3), predisposes an individual to a higher risk of diseases and premature mortality (4, 5).

In fact, evidence from developed countries suggest that the prevalence of obesity is increasing at exponential rates (6). People with obesity are at risk of heart attack and diabetes and show high level of decreases in both productivity and life expectancy (1, 7, 8). Economically, the high prevalence of obesity in most countries has led to significant increase in both direct medical costs and indirect costs from lost in productivity (9–11).

Global statistics show that the European Union (EU) has the second highest rate of overweight people (12), reaching 58% in 2014. In 2012, the total adult population in Spain who had 24.9>BMI <30 and BMI>30 were 39 and 23%, respectively (13). Consequently, about 9.7% of annual health expenditure is spent on treating overweight and obesity related diseases, the third largest number after Netherlands and Norway (14). Therefore, overweight and obesity have become major concern for governments (15).

According to Moodie et al. (16), obesity itself is a market failure given that the market allocation of goods generates economic losses for the society. To deal with this, the EU has undertaken, from 2005, over 300 initiatives in order to boost healthy nutrition and physical activity, through the creation of the *Strategy for Europe on nutrition, overweight, and obesity-related health issues* (17). However, the results of these initiatives, despite being positive, seem inadequate to transform the situation (18). This clearly suggests that the effectiveness of public policies/programs targeted at reducing the prevalence of overweight/obesity depends on clear understanding of the causative factors, which do not seem to be fully understood yet (19).

According to Cutler *et al*. (20), the reduction in the prevalence of obesity (individuals with a BMI higher than 30 kg/m^2^) is slow because obesity is influenced by many factors. Among those factors, socioeconomic ones such as income is key to explaining food consumption from the point of Classical Utility Theory and Consumer Behavior—mainly on the Lancaster Theory (21), where consumers choose whichever option offers them the maximum utility. As a result, income, household structure, education, and age are core causes of food choices (the decision to purchase a particular food product) (22, 23). From this base, the classical utility model has been extended as reflected by new models coming from the seminal work of Kahneman and Tversky (24) and Thaler (25, 26), that use cognitive psychology to understand the failure to maximize the utility of choices (27). For instance, Shepherd (23), has shown that intrapersonal factors such as attitude, beliefs and perceptions are also strong drivers of food choices and dietary behavior (decision to consume). Another key factor is perceived risk that affects every stage of the consumer decision-making process (28).

Considering the decision of food intake as an individual decision but influenced by complexity of factors, this paper attempts to investigate both the intrapersonal and socioeconomic factors influencing overweight and obesity for more targeted public policies. In order to do so, first, a conceptual model is built based on the core socioeconomic and intrapersonal factors that influence body weights. Second, the model is tested by empirically applying it to data from Catalonia (Spain) using non-linear path analysis (29, 30). It must be taken into account that studies on obesity in Spain have been limited by the approach (31), year (32), or age of respondents (33, 34). To the best of our knowledge, this analysis is the first to use risk preferences, together with socioeconomic and intrapersonal factors to study body weights. In addition, we applied a non-linear robust path analysis, which does not ignore the existence of non-linear relationships among the covariates. There is no known study exploring holistically the relationship between body weight and multiple factors such as those researched in this work. Previous studies used multiple regressions (35–37), correlations (37–39), or descriptive analysis to investigate the relationship between body weights and individual inherit factors. However, these studies do not present a comprehensive model to explore all the factors influencing overweight and obesity at a goal.

The rest of the paper will be organized as follows: Section Conceptual Framework discusses the conceptual framework. Section Research Methodology describes the data and the structural model applied to our data. Section Result and Discussions present and discuss the results generated from our data. Section Conclusion provides summary and some concluding remarks.

## Conceptual Framework

The utility-maximizing models of behavior from economic theory (15) and the Theory of Planned Behavior from psychology (40) are among the most broadly implemented theoretical frameworks to study food choices [see Salazar-Ordóñez and Rodríguez-Entrena (27)]. However, other theories such as the Steenkamp and Dekimpe (41) conceptual framework for agri-food markets have also emerged. In their framework, environmental factors, economic, and person-related factors, which are delimited as intrapersonal drivers by Shepherd (23), are highlighted. Based on the abovementioned theories, we postulate that overweight and obesity are because of food choices (15) patterned by socioeconomic and intrapersonal factors.

According to Steenkamp and Dekimpe (41), economic and sociodemographic features are drivers of the whole decision-making process with respect to food, from the recognition of the need until the choice itself. The former, drawing from Deaton and Muellbauer (42), emphasizes income as one of the main economic factors which determines food demand. Moreover, in Spain, literature finds socioeconomic factors to play important role in the development of body weights (43). However, studies on the relationship between income and obesity show mixed results. For instance, Mendez et al. (44) found a strong positive relationship between body weight and income. On the contrary, studies such as Costa-Font and Gil (45) and Nayga (46) show higher income to be associated with lower body weight. Similarly, studies on the relationship between body weights and marital status show inconclusive results. Some scholars suggest that married persons are more likely to be obese than unmarried ones (47–49). However, studies like Kittel et al. (50) suggest otherwise.

Gender and age are classified as two of the foremost biological factors which affect food choices by Steenkamp and Dekimpe (41). From the gender context, literature suggests that women tend to have bigger body mass index than men (51). A survey by Estrategia NAOS (52) found that overweight and obesity tend to increase with age among the Spanish population. Macino *et al*. (53) have highlighted that better quality of the diet and regular exercise are associated with higher income. Finally, the usual demographic variable: level of education sways the interpretation and processing of information about food (41). Empirically, Grossman (54) showed that higher educational levels decrease the prevalence of obesity. Shepherd (23) has explained that perhaps people with higher education eat healthier diets because they can obtain, process, interpret, and apply information regarding healthy diet.

Based on the above, the following hypotheses are defined:

**H1:** Overweight and obesity are influenced by income.
**H2:** Overweight and obesity are influenced by marital status.
**H3:** Overweight and obesity is higher for women.
**H4:** Overweight and obesity increases with age.
**H5:** Overweight and obesity decreases with level of schooling.

Attitude is one of the core intrapersonal factors which can be defined as the positive or negative predisposition to perform a behavior (40). Shepherd (23) points out that attitude such as personal meanings linked to foods determines nutrition behaviors. In this regard, the prevalence of negative attitudes toward obesity (ATOP) has increased by 66% over the past decade (55, 56). Some scholars believe that weight stigmatization or negative attitudes toward obese persons is a useful tool to motivate people with obesity to adopt healthier lifestyle behaviors (57). In addition, people's belief that a behavior is under volitional control, the so called perceived behavioral control, is key when explaining behaviors (40). This can be extended not only to our own behavior but also other people's behavior. Therefore, some literature finds significant and positive correlation between attitudes toward obese persons (ATOP) and beliefs about obesity (BAOP) (58). This means that individuals who have positive attitudes toward obese people believe that obesity is uncontrollable, or obesity is not under volitional control. For example, Flint *et al*. (59) assert that more negative attitudes toward people with obesity are associated with a stronger belief that obesity is controllable in the UK (60). In addition, they also show a positive relationship between belief that obesity is controllable and overweight. Therefore, the following hypotheses are defined:

**H6:** Overweight and obesity increases with positive attitude toward obesity.
**H7:** Positive attitude toward obesity decreases with beliefs about volitional control for obesity.
**H8:** Overweight and obesity increases with beliefs about volitional control for obesity.

Regarding the role of body perceptions, body image is considered a multifaceted construct that involves an individual's perceptions, thoughts, feelings, and behaviors about the size, shape, and structure of his/her body (61). There has been a rapid concern about body image over the years; the prevalence of body image dissatisfaction (BID) has increased especially among adolescents (62, 63). Literature suggests that individuals who are dissatisfied with their body are more likely to adopt behaviors that may place them at risk for more weight gain and poorer overall health (64, 65). Body image dissatisfaction is found to be strongly correlated with body weight control practices, mainly to be on diet, in both males and females (66, 67), which is a well-known cognitive strategy to combat food overconsumption (68). In addition, some people fail to control their weight because they hold wrong perceptions about it (weight perception). For example, literature indicates that overweight people perceive themselves to weigh less than their actual weight (69). Based on the above-mentioned studies, the hypotheses are:

**H9:** Body image dissatisfaction increases with overweight and obesity
**H10:** Overweight and obesity increases with the number of respondents on diet.
**H11:** Number of respondents on diet increases with body image dissatisfaction.
**H12:** Overweight and obesity decreases with correct weight perception.

Finally, risk attitudes [also considered as risk aversion, see (70)] plays a relevant role in health risks people are willing to take (15). However, losses are considered a key signal of risk since no reliable response to risk can be obtained without considering them (71). Therefore, it results that overweight and obese people are found to be less risk averse (72, 73). Indeed, Jarmolowicz *et al*. (74) show that impulsivity is higher in obese people. Meanwhile, according to Koritzky *et al*. (71), when loss aversion is applied to body weight, people may be more sensitive to a psychological loss such as gaining weight. However, these authors do not find any correlation between what they called the aversion to weight-gain (loss aversion) and people's weight. In any case, the role of loss aversion in the development of overweight and obesity has been largely unexplored. In addition, we have included the relationship between risk averssion and loss aversion to bring to bare their roles in the rise of the prevalence rates of obesity, a subject not given too much attention in literature, considering that part of risk preference involves people's willingness to obtain losses. As a result, the following hypotheses are posited:

**H13:** Higher risk aversion is associated with overweight and obesity.
**H14:** Lower loss aversion is associated with overweight and obesity.
**H15:** Loss aversed persons are also risk averse.

**Figure 1:**
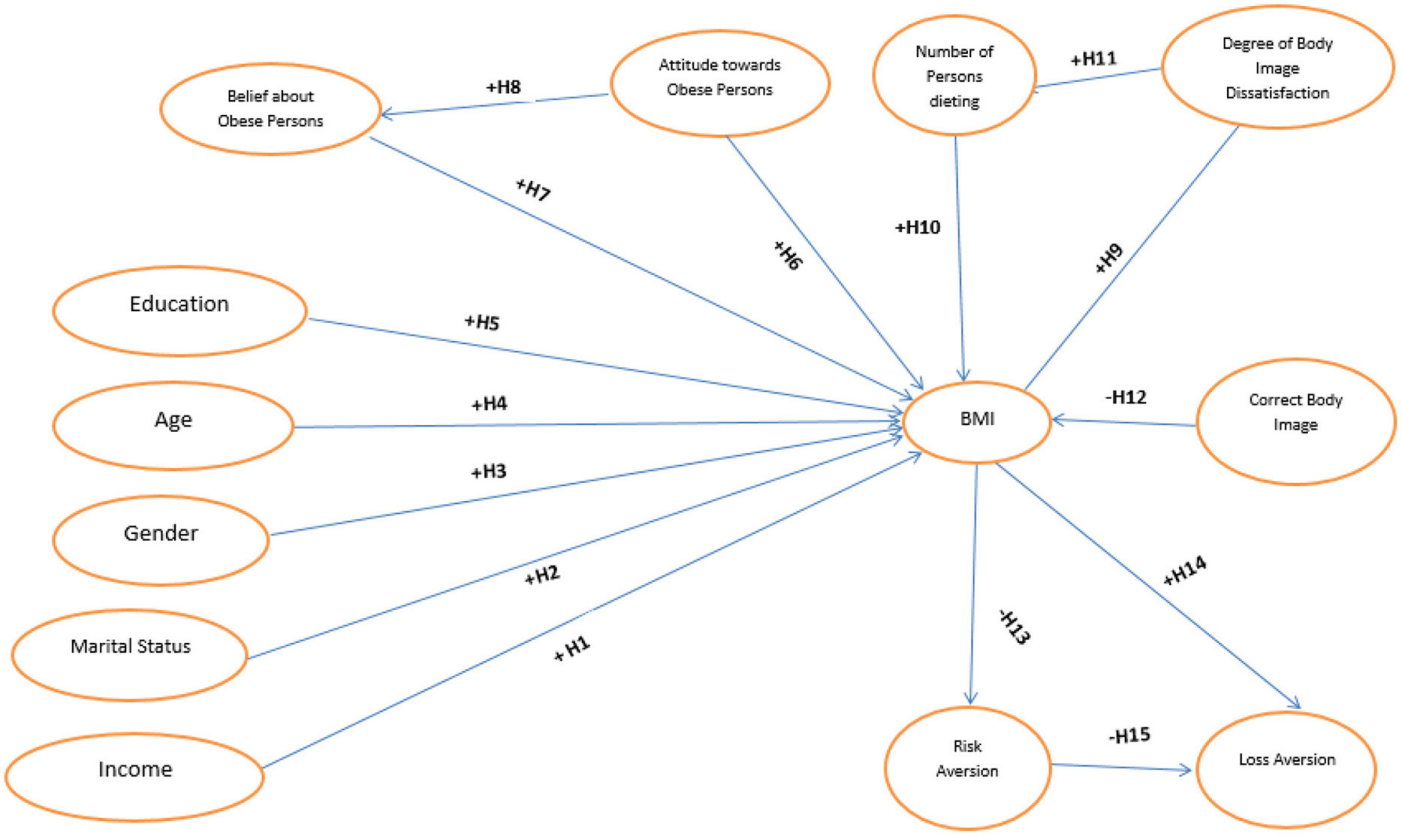
Postulated relationships between BMI, behavioral, psychological, and socioeconomic factors.

Figure 1 depicts the conceptual model that is tested here.

## Research Methodology

### Sample

The study is based on household and experimental survey carried out in Barcelona, which is located in Catalonia. Catalonia is one of the wealthiest regions in Spain; it has 30.769€ of GDP per capita in 2018, significantly higher than the average Spanish GDP per capita (25.730€), with the Metropolitan Area of Barcelona being the first area in population and the third in GDP per capita (75). A random sample of 180 individuals were surveyed^2^. However, eight surveys were discarded due to incomplete answers. The distribution of the respondents was based on the 2012 distribution of persons by BMI from the National Health Survey (76). Survey participants signed a letter of confidentiality before the start of the experiment and were paid 30 euro for completing the survey. Each participant completed the entire questionnaire on an average of 60–75 min. The survey questionnaire comprised of questions eliciting, on one section, the socioeconomic features, on the second section, the intrapersonal factors such as attitude and beliefs, and, on the third section, risk aversion and loss aversion.

### Measures

#### Weight Status Outcomes

Body weight and standing height were directly measured by providing respondents with weighing scale and stadiometer to measure their weights and heights. Body Mass Index (BMI=weight/height^2^, kg/m^2^) were calculated for each subject. Subjects were categorized into four different weight groups: as underweight (BMI < 18.5 kg/m^2^), normal weight (BMI between 18.5–24.9 kg/m^2^), overweight (BMI between 25–29.9 kg/m^2^), and obesity (BMI ≥30 kg/m^2^) (77). In our data, the percentage of individuals who were normal weight, obese, overweight and underweight are 50.58, 11.63, 35.47, and 2.33, respectively.

#### Socioeconomics

Socio-economic variables used in our analysis were income, marital status, gender, age, and the level of schooling. First, respondents were grouped based on their income range, so, people earning gross income below 1,500 euro were assigned the value of one and zero for all other income levels. Similarly, marital status of respondents was categorized into two: married and unmarried. Married people were assigned the value of one and zero if otherwise. Third, gender was measured as a categorical variable where females were assigned the value of one and zero if otherwise. Age was a continuous variable, defined as the age of the respondent at the time of the data collection. Finally, schooling level was categorical, defined as one if the household head has attained University education and zero if otherwise.

#### Attitudes and Beliefs

Attitudes toward obesity (ATOP) and beliefs about obesity (BAOP) scales were developed in 1991 (78). The estimates of ATOP and BAOP show the extent of individuals' attitudes (positive or negative) and belief (positive or negative) about obesity. ATOP scores range from 0-120 across 20 items; where low (high) scores represent negative (positive) attitudes toward people with obesity (see Appendix A). To calculate each respondent's ATOP score, three steps were followed. First, responses to the following items were multiplied by −1 (i.e., reverse the direction of scoring): Item 2 through Item 6, Item 10 through Item 12, Item 14 through Item 16, Item 19 and Item 20. Second, the responses to all items were added up. Finally, a value of 60 was added to the value obtained in Step 2. Higher ATOP score numbers indicated more positive attitudes. Similarly, BAOP scores also ranged from 0-48 across 8 items as shown on Appendix B; where low (high) scores represent a stronger (lesser) belief that obesity is controllable, i.e., volitional control. To calculate each respondent's BAOP score, we also followed three steps: Step 1: Multiply the response to the following items by−1 (i.e., reverse the direction of scoring): Item1, Items 3 through Item 6, Item 8. Step 2: Sum the responses to all items. Step 3: Add 24 to the value obtained in Step 2. This value is the BAOP score. Higher BAOP score numbers indicated higher belief that obesity is uncontrollable.

#### Body Image Satisfaction and Weight Perception

**Figure 2:**
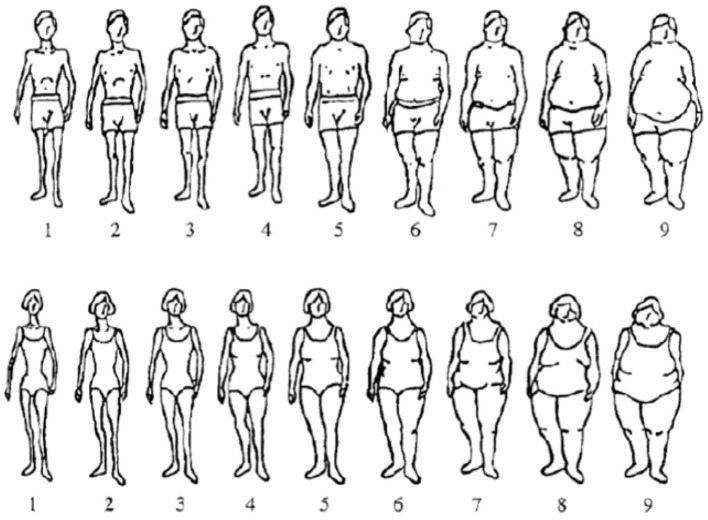
Stunkard scale. Adapted from Stunkard *et al.* (82).

To determine individuals body image satisfaction, the Stunkard scale (79) was used after a thorough review. The reliability of the Stunkard scale has been confirm in social science research (80, 81). The Stunkard scale in Figure 2 presents visual figures that represent nine gender-specific body-shape silhouettes ranging from very thin (assigned a value of 1) to very big (assigned a value of 9).

Respondents were asked to choose from the nine body shapes which silhouette best represented their “current shape” and then their “preferred shape.” We classified the Stunkard figure rating scale (SFRS) figures as underweight (body shapes 1, 2), normal weight (body shapes 3, 4), overweight (body shapes 5–7), and obese (body shapes 8, 9). The difference between perceived current body shape and preferred body shape was used to determine the degree of body image dissatisfaction. Values approaching zero reflect less discrepancy (i.e. the respondent choses the same figure to represent their current size and their ideal size). Based on the results from Figure 2, participants were classified into three groups: (1) satisfied with current body shape (current = preferred); unsatisfied with their body image: (2) desired to be thinner (current > preferred), and (3) desired to be heavier (current < preferred). We also considered weight misperception among our respondents based on the variation between subject's choice of “current weight” and their measured weight status. If the individual's current weight from the Stunkard scale was equal to the measured BMI, then the individual had the correct perception about their weight. However, if the measured BMI was higher (or lower) than the figure chosen on the Stunkard scale as the current image then the individual had a wrong perception about their weight. Thus, negative and positive scores indicated that the individuals perceived themselves as thinner or weightier than the ideal, respectively, whereas a zero-score indicated correct weight perception. Finally, we created a categorical variable for “persons on diet,” where an individual is assigned the value of one if he/she followed a strict diet for weight control purposes and zero if otherwise.

#### Risk and Loss Aversion

Respondents elicited their risk and loss aversion coefficients through incentivised lotteries. The goal of using lotteries was to be able to elicit the true behavior of consumers for monetary gains and losses. According to Koritzky *et al*. (71), the parameters estimated from monetary choices have similar characteristics to those from weight-gain, so some of the same underlying mechanisms may be determined both loss aversion (for monetary values) and weight gain. The experimental procedure used was based on the seminal work of Tanaka *et al*. (83). Individual's utility function indicating their risk and loss aversion were modeled following the Prospect Theory (PT) framework (24). Mathematically, the utility function following the prospect theory framework can be expressed as follows:

(1) $$\begin{array}{l} PT\left(x,y;p\right)=pv\left(x\right)+\left(1-p\right)v\left(y\right) \end{array}$$

(2) $$\text{where}v\left(x\right)=\{\begin{array}{ll} x^{\sigma } & for\;x\geq 0\\ -\lambda \;(-x^{\sigma }) & for\;x<0 \end{array}\;$$

(3) $$\text{and }w\left(p\right)=exp\left[-{\left(-\ln p\right)}^{\gamma }\right]$$

*PT* (*x, y*; *p*) is the expected prospect value over binary prospects consisting of the outcome (*x, y*) with the corresponding probability (*p*, 1 – *p*). In our experiment, (*x, y*; *p*) is specified for plan A and plan B in all scenarios. Note that the value function *v*(*x*) should be estimated with *x*^σ^ for *x* > 0 or –λ (–*x*^σ^) for *x* < 0. The parameter σ represents concavity of the value function (risk aversion)—high values indicate respondents are risk loving, λ represents the degree of loss aversion—high values indicating respondents are more loss averse, and γ is a proxy for the non-linear probability weighting.

To elicit the three PT parameters (σ, λ, and γ) in equations (1–3), respondents were given three series of games that contained 35 pair-wise choices. Appendix C shows the three series of games consisting of plan A and plan B. Series 1 consists of 14 pairwise games. Series 2 consists of 14 pairwise games, and Series 3 consists of seven pairwise games. Each respondent had three options: (a) choosing Plan A throughout all games; (b) choosing Plan B throughout all games; and (c) choosing Plan A for a certain number of games and then switched to Plan B for the rest. Individuals who were more averse to loss would choose Plan A a greater number of times over Plan B in both series 1 and 2. The switching points in series 1 and series 2 were used to calculate the average risk aversion and probability weighting parameter (83). Derived risk aversion estimates^3^ are shown in Appendix C. Based on the risk aversion estimates individuals can be categorize as being risk averse (*if σ* <1), risk neutral (*if σ*=1) and risk loving (*if σ*>1). The loss aversion parameter was calculated by formulating inequalities involving the switching points in Series 3 (83). Similarly, for the loss aversion estimates, individuals were either loss averse (σ≥1) or not (σ <1).

### Data Analysis

Structural equation modeling (SEM) was first introduced by Wright (85) by studying relationships between variables represented in a “path diagram” and later became known as “path analysis.” Path analysis is an advanced statistical technique used to examine how exogenous and endogenous variables affect each other in the path model (86). Path analysis starts with a theory to formulate a structural model (path diagram) that provides a pictorial representation of relationships among variables (87, 88). Calculation of path estimates provides the degree and direction of effects that is postulated to exist among a set of variables (86, 89). This technique has been applied in different field of knowledge such as the area of technology integration (90, 91), quality practices in business (92), career development (93), and reasoning abilities (94). Kock (95) proposed the use of robust non-linear path analysis that exhibit certain advantages over previous models [(96); McDonald, 1996; (97)]. The use of the robust path analysis is computationally simpler, distribution-neutral, and a more reliable approach compared to previous path analysis techniques (30).

Considering the complexity of factors that influence overweight and obesity, we applied the robust path analysis to estimate path coefficients instead of the traditional SEM analysis for two main reasons. First, robust path analysis does not impose linear restrictions on the parameters. Second, all *p*-values can be estimated through distribution-neutral nonparametric procedures. This is important, due to the non-normal distribution of some variables used in our model.

Robust path analysis can use both standardized (zero mean and 1 standard deviation) and/or non-standardized variables for the estimation of path coefficients. For ease of interpreting our results, standardized values were used in our estimation. We followed the steps proposed by Iriondo *et al*. (98) to estimate our conceptual model. First, hypotheses formulated based upon a priori knowledge are translated into series of equations to be estimated. Second, data on all observable variables defined in the model were collected from the population of interest. Third, we applied the robust non-linear multivariate regression to estimate path parameters. We conclude by testing the global goodness of fit as well as the detailed goodness of fit of the estimated model using predetermined criteria. Where goodness of fit test is rejected, the path model is modified and re-estimated to improve the goodness of fit without compromising on the underlying theory.

Robust non-linear path analysis (29, 30) produces outputs for model fit and quality assessment, test for multicollinearity and generate predictive validity tests. Overall model fit was assessed using the Average path coefficient (APC), the Average R-squared (ARS) and the Average adjusted R-squared (AARS). When APC, ARS, and AARS have coefficients with *p* < 5%, it indicates satisfactory fit to the data. The Average block VIF (AVIF) and Average full collinearity VIF (AFVIF) were used to test for vertical and full collinearity based on conservative multivariate data analyses criteria. Variance inflation factors of 3.3 or lower suggest the existence of no vertical collinearity in a variable block (96, 99). Other predictive validity test includes Tenenhaus GoF (GoF), Sympson's paradox ratio (SPR), R-squared contribution ratio (RSCR), Statistical suppression ratio (SSR), and Non-linear bivariate causality direction ratio (NLBCDR).

The magnitude and sign of the path coefficients are estimated taking into account non-linearity that may exist among some variables. This is an important feature because some variables may exhibit non-linear relationships with body mass index which is important to consider (29). The magnitude and sign of path coefficients indicate the degree and direction of effects that exist among a set of variables (86).

## Results

### Descriptive Statistics

Non-standardized averages and frequencies of the variables that were used in the path analysis are shown in Table 1. Individuals who earned gross income <1500 euros represented 32% of the total sample, indicating that majority of the respondents earn more than 1,500 euro per month. About 70 and 69% of the respondents were female and married, respectively. Our data also shows that the average individual in our sample is within the middle age category, with an average age of 46 years. Those with only University education represent 36% of the total sample. In addition, only 24% of the respondents followed a strict diet. Regarding the risk preferences, individuals are risk averse and more averse toward losses. Even though, the average BMI (25.17) indicate an overweight population. As a result, about 73% of the respondents were dissatisfied with their body showing an average degree of body image dissatisfaction of 1.93.

**Table 1:** Socioeconomic description of the sample.

**Sociodemographic characteristics**	**Percentages**			
Gender (Female = 1)	70			
Marital status (Married = 1)	69			
Education (University = 1)	36			
Income Levels (<1,500 = 1)	32			
Number of respondents on Diet	24			
	**Mean**	**Min**	**Max**	**Std. Dev**.
Age	45.80	20.00	70.00	11.22
Average risk aversion coefficient	0.58	0.00	1.50	0.37
Average loss aversion coefficient	3.67	0.00	11.79	3.88
Body mass index	25.17	17.53	46.24	4.21
Degree of body image dissatisfaction	1.19	0.00	6.00	1.07
Belief about Obese People (BAOP)	21.65	11.00	41.00	4.31
Attitude toward Obese Persons (ATOP)	65.33	18.00	110.00	14.93

### Model Estimates

The overall model^4^ fit was tested based on the significance of the Average Path Coefficient (APC), Average R-squared (ARS), and Average adjusted R-squared (AARS). Table 2 shows that these indices are significant at *p* < 0.05 indicating a well-fitted model. The Average Variance Inflation Factor (AVIFs) and Average full collinearity VIF (AFVIF) coefficients suggest the path model is free of multicollinearity at the variable level and the entire model. In addition, the Goodness of Fit (GoF) index suggests that the overall goodness-of-fit level between model and data is large. The Non-linear bivariate causality direction ratio (NLBCDR) was 0.96, which is greater than the acceptable value of ≥ 0.7 (95).

**Table 2:** Model fit and quality indices.

**Index**	**Value**	**Interpretation**
Average path coefficient (APC)	0.214	<0.01
Average R-squared (ARS)	0.141	<0.05
Average adjusted R-squared (AARS)	0.131	<0.05
Average block VIF (AVIF)	1.123	Acceptable if ≤ 5, ideally ≤ 3.3
Average full collinearity VIF (AFVIF)	1.370	Acceptable if ≤ 5, ideally ≤ 3.3
Tenenhaus GoF (GoF)	0.376	Small ≥ 0.1, medium ≥ 0.25, large ≥ 0.36
Sympson's paradox ratio (SPR)	1.000	Acceptable if ≥ 0.7, ideally = 1
R-squared contribution ratio (RSCR)	1.000	Acceptable if ≥ 0.9, ideally = 1
Statistical suppression ratio (SSR)	0.800	Acceptable if ≥ 0.7
Non-linear bivariate causality direction ratio (NLBCDR)	0.900	Acceptable if ≥ 0.7

*The threshold values can be seen in (30, 95)*.

#### Socioeconomic Factors

**Figure 3:**
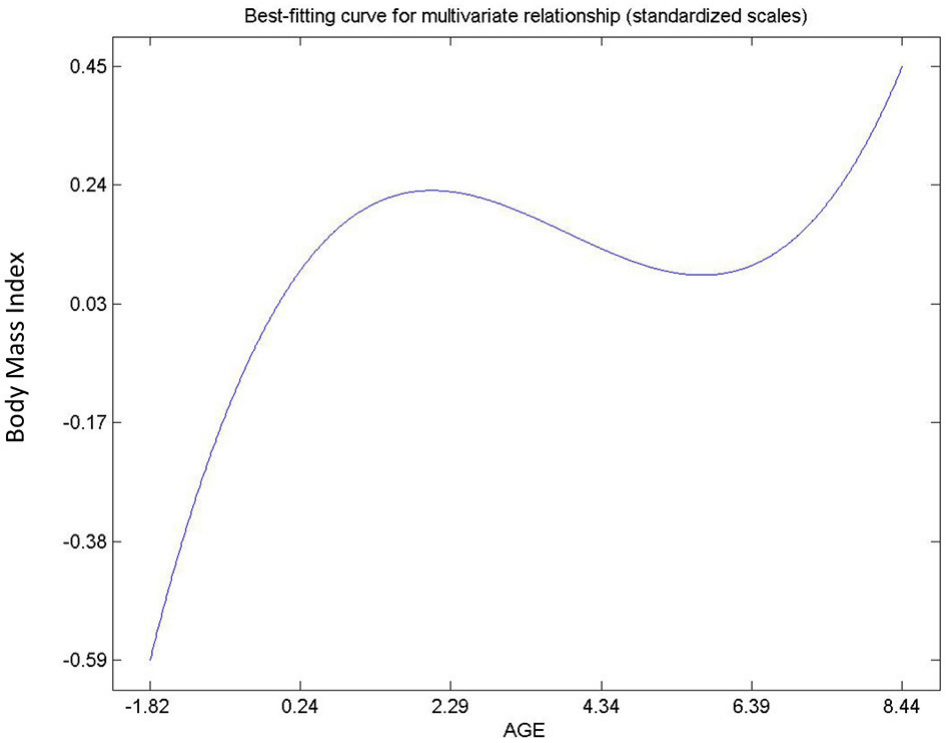
Relationship between age and BMI.

The summary of the hypotheses is displayed on Table 3. All socioeconomic variables (H1, H2, H3, and H4) have significant impact on Body Mass Index except level of schooling (H5). The variable with the largest impact is gender whilst that with the least impact is income level. The relationships between income level, marital status, gender, and BMI are linear. However, Figure 3 suggest an inverted “S” curve relationship between age and BMI. This suggest that BMI increases (positive slope) with age until an inflection point where it begins to decrease (negative slope) but rises (positive slope) again. This type of relationship suggests middle-aged groups tend to have a negative relationship with BMI.

**Table 3:** Description of hypotheses relating drivers of body mass index.

**Hypotheses**	**Relationships**	**Expected sign**	**parameter**	**Standard error**	**Effect size**
**H1**	Overweight and obesity are influenced by income.	+	0.12^*^	0.074	0.010
**H2**	Overweight and obesity are influenced by marital status.	+	0.14^**^	0.074	0.016
**H3**	Overweight and obesity increases for women.	+	−0.21^***^	0.073	0.032
**H4**	Overweight and obesity increases according to age.	+	0.18^***^	0.073	0.041
**H5**	Overweight and obesity decreases according to level of schooling.	–	−0.10^*^	0.075	0.015
**H6**	Overweight and obesity increases according to positive attitude toward obesity	+	0.26^***^	0.072	0.063
**H7**	Positive attitude toward obesity decreases by beliefs about volitional control for obesity.	+	0.33^***^	0.071	0.109
**H8**	Overweight and obesity increases according to beliefs about volitional control for obesity	+	0.14^**^	0.074	0.019
**H9**	Body image dissatisfaction increases according to overweight and obesity	+	0.69^***^	0.066	0.479
**H10**	Overweight and obesity increases according to people on diet at household level.	+	0.25^***^	0.072	0.065
**H11**	People on diet at household level increases according to body image dissatisfaction	+	0.20^***^	0.073	0.040
**H12**	Overweight and obesity decreases according to right weight perception.	–	−0.12^*^	0.074	0.021
**H13**	Risk aversion decreases according to overweight and obesity	+	0.16^***^	0.074	0.025
**H14**	Loss aversion increases according to overweight and obesity.	+	−0.09*n*.*s*.	0.075	0.0008
**H15**	Loss averse increases according to risk averse.	–	−0.21^***^	0.073	0.043

*** p < 0.01;

** p < 0.05;

* *p < 0.1; n.s.- non-significant—based on a two-tailed t-test for t_(4999)_ from a bootstrapping technique. According to Cohen (100), f size values of 0.02, 0.15, and 0.35 result in small, medium and large effects, respectively*.

Since lower income and less educated groups are more overweight and obese than the rest of the population, public policies should focus on making nutrient dense foods affordable and accessible through the use of subsidies and coupons. Educational campaigns should also be targeted at poor communities.

#### Intrapersonal Factors: Attitude and Beliefs

**Figure 4:**
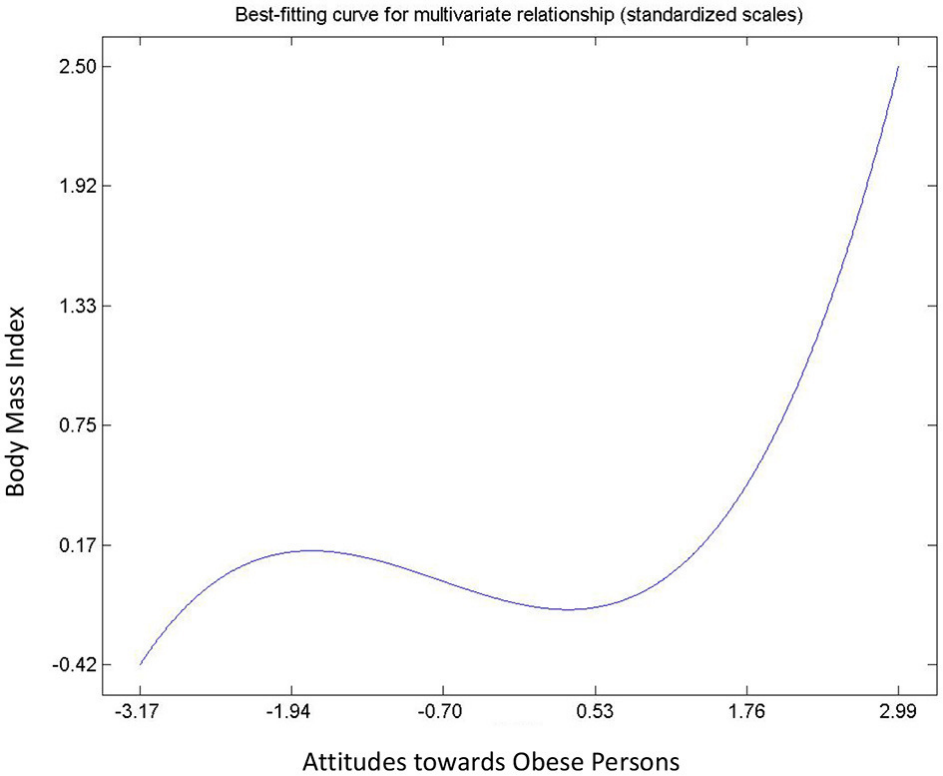
Relationship between attitudes toward obesity and BMI.

**Figure 5:**
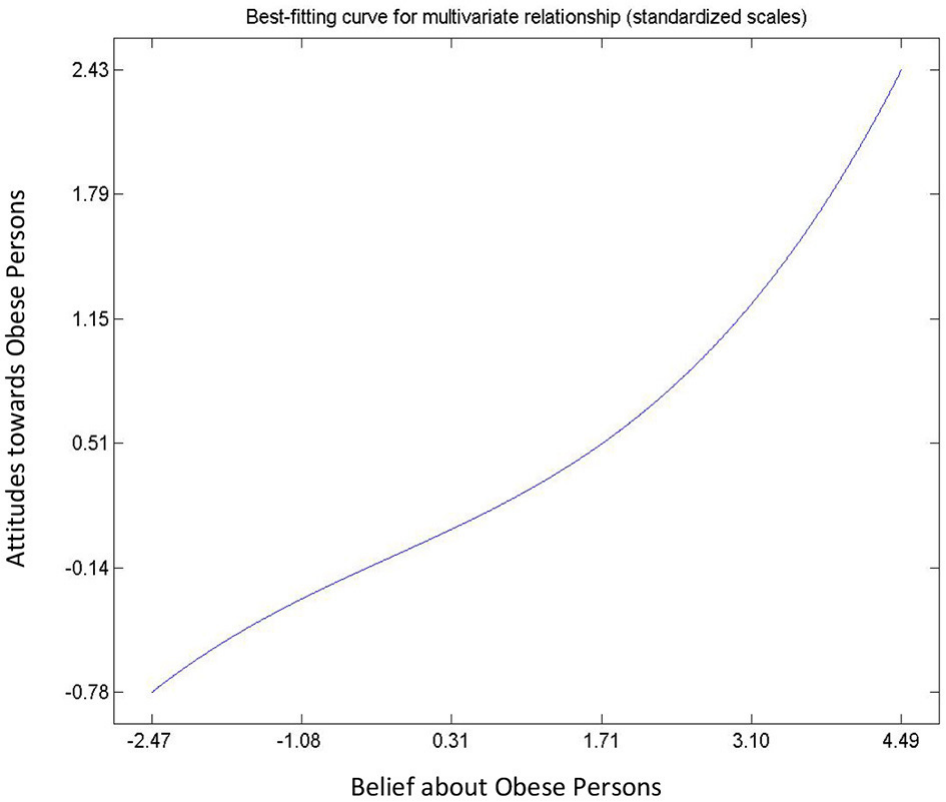
Relationship between people attitude and beliefs toward obesity.

**Figure 6:**
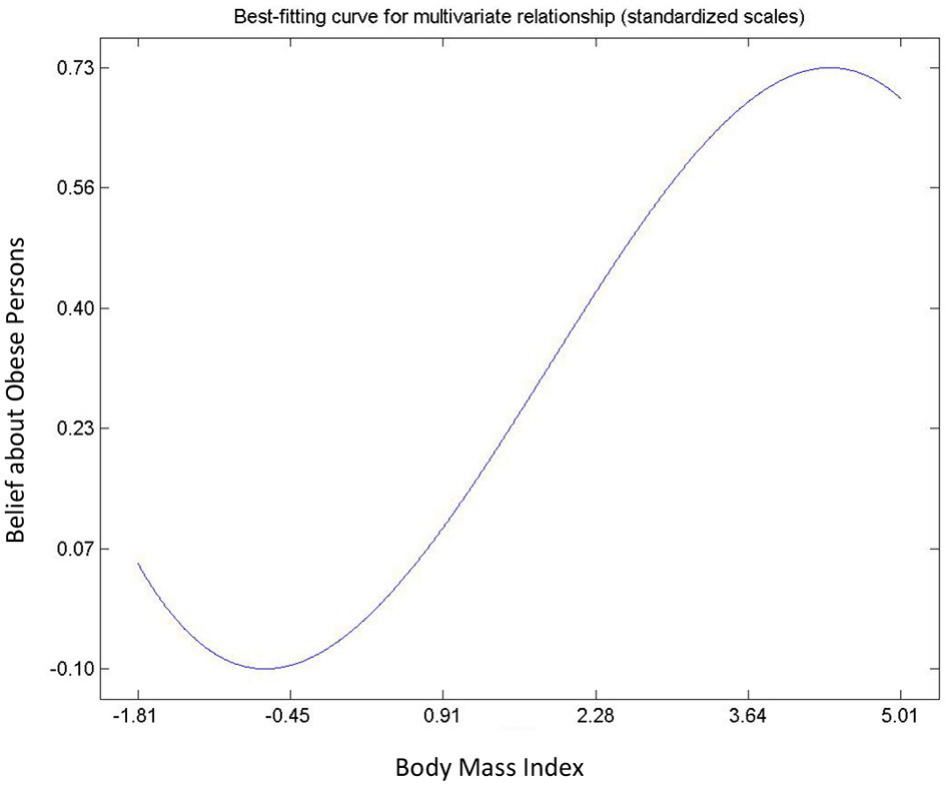
Relationship between belief about obesity and BMI.

Hypothesis 6, 7 and 8 describe the relationships between attitude and beliefs about obesity, and BMI. All relationships were found to be significant (Table 3) and non-linear (Figures 4–6). Hypothesis 6 suggests that a person's negative attitude toward obese people will increase as their own BMI reduces. However, further examination suggest that peoples' attitude and BMI show the existence of “S” curve relationship (see Figure 4). In general, hypothesis 7 suggests that a person who believes that obesity is controllable exhibits negative attitude toward obese persons. On the contrary, Figure 5 shows that a person's belief and attitude have an exponential relationship. This suggests that a standard deviation increase in belief that obesity is uncontrollable will lead to more than proportionate increase in positive attitudes. Finally, an increase in BMI will increase the belief of the average respondent that obesity is uncontrollable, so it is not under volitional control (Hypothesis 8). However, Figure 6 shows that the true relationship is an “S” curve.

#### Intrapersonal Factors: Perceptions - Body Image Dissatisfaction and Weight Perception

**Figure 7:**
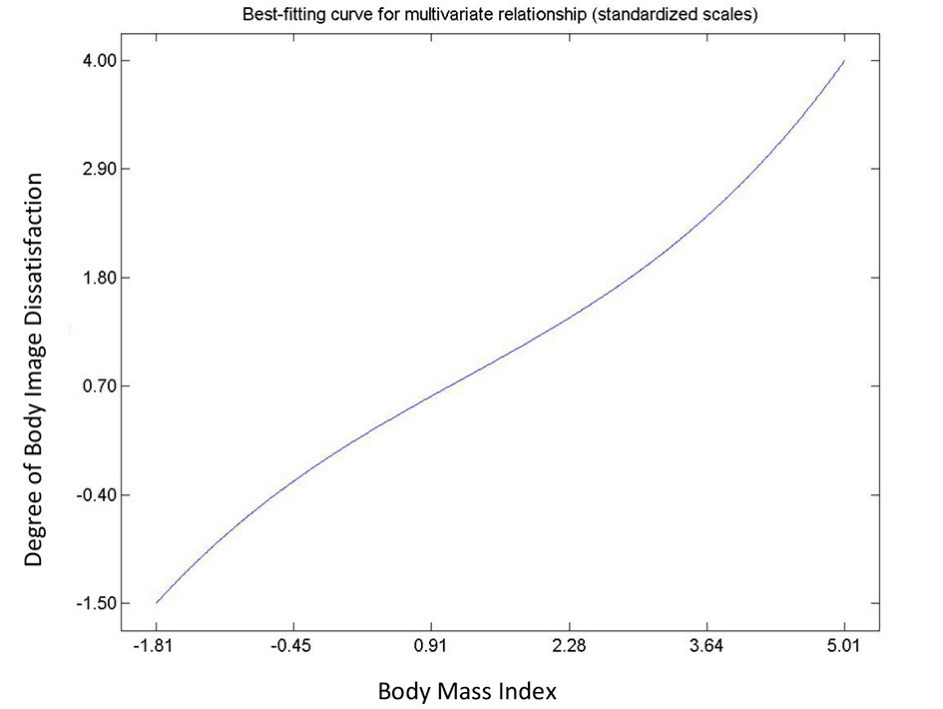
Relationship between degree of body image dissatisfaction and BMI.

**Figure 8:**
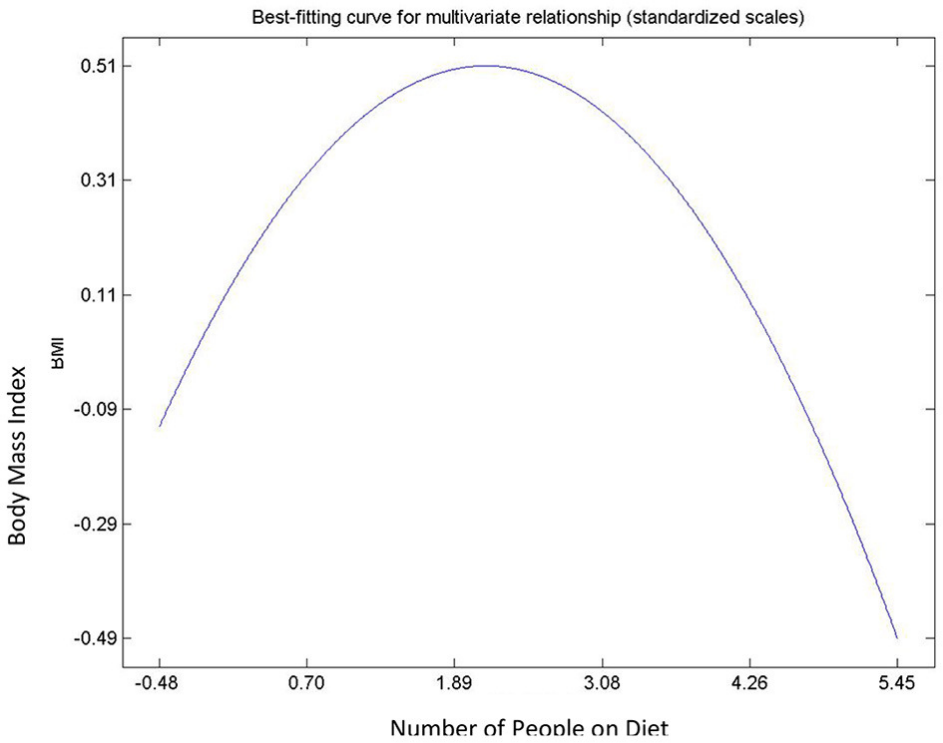
Relationship between number of people on diet and BMI.

**Figure 9:**
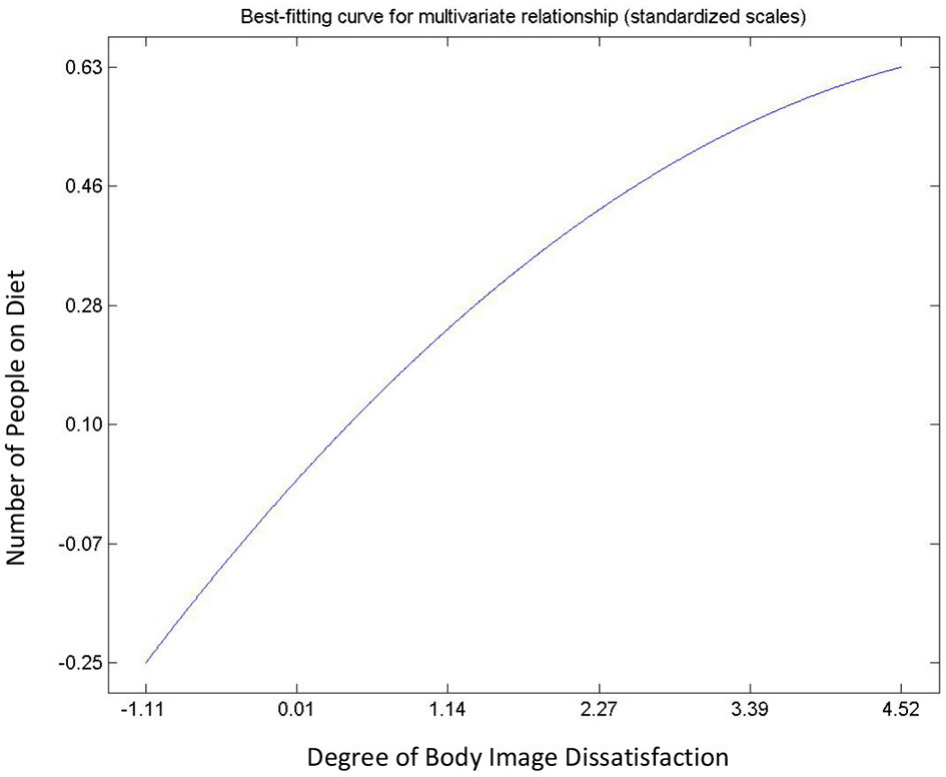
Relationship between number of people on diet and body image dissatisfaction.

Hypothesis 9, 10, 11, and 12 describes the relationships between body image dissatisfaction and weight perceptions (including number of subjects on diet), and BMI. All postulated relationships were significant and positive. With the exception of the relationship between the number of people with correct body image, i.e., correct weight perception, and BMI (Hypothesis 12), the rest were non-linear. There was an exponential, an inverted “U” curve, and exponential relationships for the degree of body image dissatisfaction and BMI (Figure 7); number of people on diet and body mass index (Figure 8); and the people on diet and degree of body image dissatisfaction (Figure 9), respectively. In general, hypothesis 9 supports that a person's degree of body image dissatisfaction will increase as his/her BMI increases. Similarly, the average number of people on diet in the population will increase as BMI and the average degree of body image dissatisfaction increases (hypothesis 10 and hypothesis 11). Hypothesis 12 suggests that the average number of people with correct body image will reduce as the average body mass index of respondents' increases.

#### Intrapersonal Factors: Perceptions - Risk and Loss Aversion

**Figure 10:**
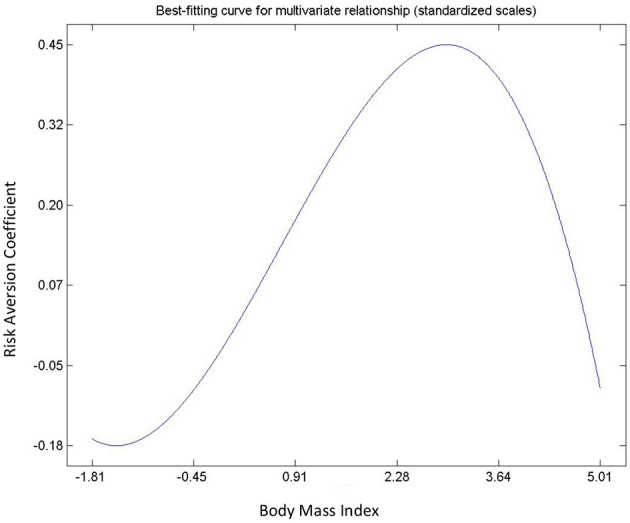
Relationship between risk aversion and BMI.

**Figure 11:**
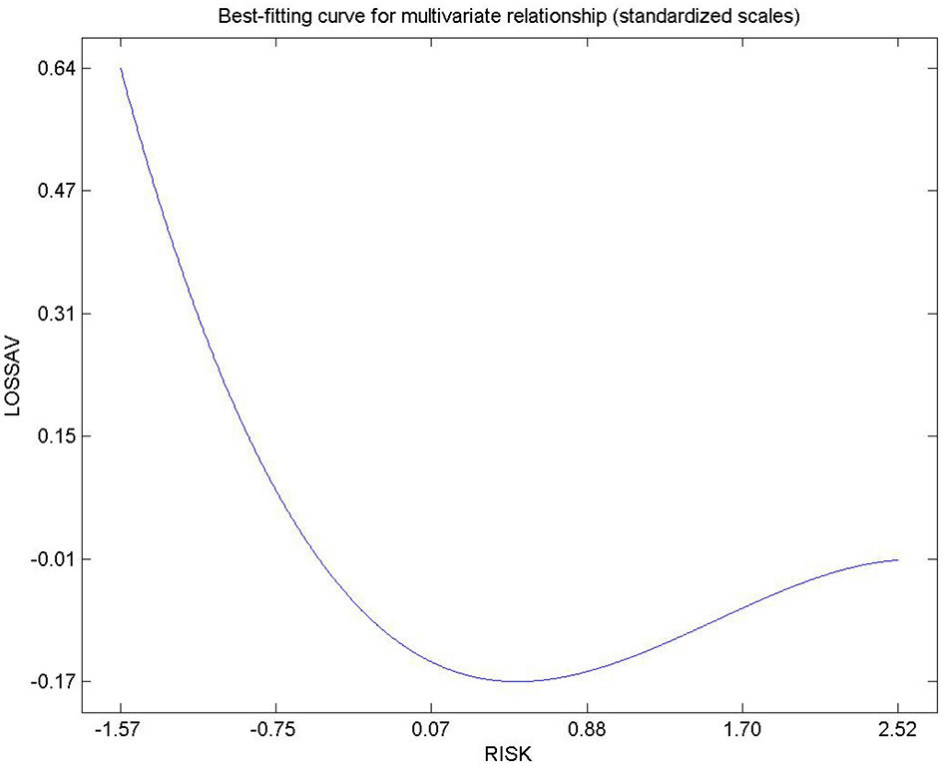
Relationship between loss aversion and risk aversion.

Hypothesis 13, 14, and 15 were used to describe the relationships determined by risk and loss aversion and body mass index (see Table 3). The relationship between loss aversion and BMI was not significant. The remaining two hypotheses (13 and 15) exhibited non-linear relationships. Risk aversion tends to increase as BMI decreases. The relationship between risk aversion and loss aversion was positive suggesting that risk lovers are often more averse toward losses. From the context of non-linear relationships, risk and BMI exhibited an inverted “U” curve relationship (Figure 10) whilst loss aversion and risk aversion exhibited an asymmetric “J” curve relationship (Figure 11).

## Discussions

Different implications emerge from this study. First, income, marital status, gender, and age were socioeconomic factors that significantly affected BMI, i.e., overweight and obesity. Just like Costa-Font and Gil (43), we provide empirical evidence that the foremost economic variable from maximizing-utility models, income level, plays a role in overweight and obesity in the sense that incomes over 1.500 Euro match with less BMI. However, this is an opposite result with respect to that by Mendez *et al*. (44); while Ball and Crawford (101) point out that income is not a consistent variable associated with weight gain. Our finding on household structure by means of marital status supports strands of studies that found married people to have higher weight than unmarried ones (47–49). Similarly, our result also confirms previous literature in Catalonia that finds females to have lower BMI than males (102). In addition, the positive relationship between age and BMI confirms the findings of Estrategia NAOS (52), where older people tend to be more overweight and obese. However, our path model reveals that the relationship is non-linear. This non-linearity has also been confirmed by Aranceta *et al*. (103) which show that obesity increases with age in men and women: lower (5.3%) in those between 25 and 34 years but higher (26.3%) in the age group 55–60 years. Similarly, higher prevalence of overweight and obesity is confirmed by Macino *et al*. (2004) or Grossman (54); although, according to Ball and Crawford (101), there is a lack of consistency by this variable. Since lower income and less educated groups are more overweight and obese than the rest of the population, public policies should focus on making nutrient dense foods affordable and accessible through the use of subsidies and coupons. Educational campaigns should also be targeted at poor communities. We also recommend that men and older people are encouraged to engage in exercise. This can be achieved through the national sports for all program currently being implemented in Spain. Marriage is crucial in the fight against the prevalence of overweight and obesity. We believe that weekend cooking programs by top chefs and dieticians on national television can provide the avenue for changing/improving family diets.

Referring to intrapersonal factors, the significant positive relationship between attitude toward obesity and BMI supports that weight stigma is a major driver of weight gain (104). Moreover, our findings are also in line of those by Flint and Snook (60), showing that people's belief about the controllability of obesity reduces as their BMI increases; so, overweight and obese people tend to believe that obesity is not under volitional control. In addition, the general belief about obesity uncontrollability leads to positive attitudes toward obese people, as it is found by Allison *et al*. (78). However, the non-linear nature of the relationship suggests that people's positive attitude increase more than proportionate increase in the belief that obesity is uncontrollable.

According to our model, body image dissatisfaction increases with BMI, which means that overweight and obese people tend to be more discontent with their bodies, which is supported by the findings of Ålgars *et al*. (105) and Weinberger *et al*. (106). In addition, the prevalence of dieting rises with increasing BMI; thus, it can be stated that overweight and obese people go more on a diet (107), which makes sense. Indeed, body image dissatisfaction rises with the prevalence of dieting, confirming that the relationship is non-linear (108). Finally, the negative relationship between BMI and the prevalence of correct weight perception strongly confirms that overweight/obese people perceive themselves to weigh less than their actual weight (69). Societal education against prejudice toward overweight and obese persons should be encouraged in schools and television since negative body image is an important factor in the fight against obesity and overweight. In general, increase in public awareness about correct body weights and the relationship between overweight and non-communicable diseases is important since this will induce consumers to practice healthy dieting

Our finding supports that growth in body mass index and risk aversion move in opposite direction (72, 73, 109). This suggest that obese and overweight persons are likely to be risk loving (at the extreme end) whilst underweight and normal weight persons are risk averse. The negative relationship between risk aversion and loss aversion bring to light that risk averse consumers are also averse toward loses. Nonetheless, the role of risk attitudes and loss aversion requires further investigation to ascertain the use of these factors in the fight against overweight and obesity.

## Conclusion

This study conducted experiment in Catalonia, Spain to investigate how intrapersonal and socioeconomic factors affect body weights. We achieved this by developing a conceptual model using robust non-linear path modeling technique.

Our results suggest that several factors affecting body weights are non-linear in nature. These were categorized into socioeconomic and intrapersonal factors. From the policy context, our results do not support a one-for-all policy to tackle obesity since the factors affecting BMI differ between individuals even in the same county.

We, therefore, propose that government policies should be comprehensive, i.e., should targeted at different groups of individuals based on their socioeconomic characteristics, attitudes, beliefs and perceptions about body weights. For instance, policy interventions focused on behavioral changes should be targeted at the younger population.

It is worth mentioning that this study has some limitations. First, our data size is small and geographically limited. This conditions the generalization and potential of transferability of the results. Second, genetic factors and physical activities that play important role in overweight and obesity were omitted from our analysis. Third, only adult population were sampled indicating that result cannot be generalized to children and teenagers. Finally, our model does not consider the existence of bidirectional relationships between our variables.

## Data Availability Statement

The data that support the findings of this study are available on request from the corresponding author. The data are not publicly available due to privacy restrictions.

## Ethics Statement

The studies involving human participants were reviewed and approved by Center for Agro-food Economics and Development. The patients/participants provided their written informed consent to participate in this study.

## Author Contributions

WD: Developed the research idea and modeling. MS-O: Review the model and the results. JG: As a supervisor, he worked together with WD to develop the model and review the entire manuscript. All authors contributed to the article and approved the submitted version.

## Conflict of Interest

The authors declare that the research was conducted in the absence of any commercial or financial relationships that could be construed as a potential conflict of interest.

## Footnotes

^1^According to WHO, the acceptable range of Body Mass Index for adults over 20 years old is 18.5 > BMI < 24.5 kg/m2, i.e. normal weight (see http://www.euro.who.int/en/health-topics/disease-prevention/nutrition/a-healthy-lifestyle/body-mass-index-bmi).

^2^The sample size was based on the cost and timeliness of the experiment. Experimental data are very expensive and time consuming. As a result, only 180 households were sampled. However, we made sure the sample was evenly distributed according to the population distribution in Catalonia

^3^A full description of the experiment and derivation of the parameter estimates has been provided in Dogbe and Gil (84).

^4^The WarpPLS of Kock and Gaskins (30) was used to estimate the path coefficients. The outer model analysis algorithm used was the “Robust Path Analysis”
