# Supplementary material for: Disentangling the Drivers of Obesity: An Analytical Framework Based on Socioeconomic and Intrapersonal Factors
Source: Front Nutr. 2021 Mar 3;8:585318. doi: 10.3389/fnut.2021.585318 (PMC8006933; doi:10.3389/fnut.2021.585318)
Supplement: Supplementary file 1 [file Presentation_1.pdf]

Appendix A: **Attitudes Toward Obese Persons Scale (ATOP)**

Please mark each statement below in the left margin, according to how much you agree or disagree with it. Please do not leave any blank. Use the numbers on the following scale to indicate your response. Be sure to place a minus or plus sign (- or +) beside the number that you choose to show whether you agree or disagree.

-3	-2	-1	+1	+2	+3
**I strongly disagree**	**I moderately disagree**	**I slightly disagree**	**I slightly agree**	**I moderately agree**	**I strongly agree**

1. _______Obese people are as happy as nonobese people.
2. _______Most obese people feel that they are not as good as other people.
3. _______Most obese people are more self-conscious than other people.
4. _______Obese workers cannot be as successful as other workers.
5. _______Most nonobese people would not want to marry anyone who is obese.
6. _______Severely obese people are usually untidy.
7. _______Obese people are usually sociable.
8. _______Most obese people are not dissatisfied with themselves.
9. _______Obese people are just as self-confident as other people.
10. _______Most people feel uncomfortable when they associate with obese people.
11. _______Obese people are often less aggressive than nonobese people.
12. _______Most obese people have different personalities than nonobese people.
13. _______Very few obese people are ashamed of their weight.
14. _______Most obese people resent normal weight people.
15. _______Obese people are more emotional than nonobese people.
16. _______Obese people should not expect to lead normal lives.
17. _______Obese people are just as healthy as nonobese people.
18. _______Obese people are just as sexually attractive as nonobese people.
19. _______Obese people tend to have family problems.
20. _______One of the worst things that could happen to a person would be for him to become obese.

Appendix B: **Beliefs About Obese Persons Scale (BAOP)**

Please mark each statement below in the left margin, according to how much you agree or disagree with it. Please do not leave any blank. Use the numbers on the following scale to indicate your response. Be sure to place a minus or plus sign (- or +) beside the number that you choose to show whether you agree or disagree.

-3	-2	-1	+1	+2	+3
**I strongly disagree**	**I moderately disagree**	**I slightly disagree**	**I slightly agree**	**I moderately agree**	**I strongly agree**

1. _____ Obesity often occurs when eating is used as a form of compensation for lack of love or attention.

2. _____ In many cases, obesity is the result of a biological disorder.

3. _____ Obesity is usually caused by overeating.

4. _____ Most obese people cause their problem by not getting enough exercise.

5. _____ Most obese people eat more than nonobese people.

6. _____ The majority of obese people have poor eating habits that lead to their obesity.

7. _____ Obesity is rarely caused by a lack of willpower.

8. _____ People can be addicted to food, just as others are addicted to drugs, and these people usually become obese.

Allison, D.B., Basile, V.C., and Yuker, H. E. (1991). The measurement of attitudes toward and beliefs about obese persons. *International Journal of Eating Disorders, 10*, 599-607.

Appendix C: Experimental Lotteries to Risk attitudes

Series 1	Plan A	Plan B
1	4€ Yes ①②③<br>1€ Yes ④⑤⑥⑦⑧⑨⑩	6,8€ Yes ①<br>0,5€ Yes ②③④⑤⑥⑦⑧⑨⑩
2	4€ Yes ①②③<br>1€ Yes ④⑤⑥⑦⑧⑨⑩	7,5€ Yes ①<br>0,5€ Yes ②③④⑤⑥⑦⑧⑨⑩
3	4€ Yes ①②③<br>1€ Yes ④⑤⑥⑦⑧⑨⑩	8,3€ Yes ①<br>0,5€ Yes ②③④⑤⑥⑦⑧⑨⑩
4	4€ Yes ①②③<br>1€ Yes ④⑤⑥⑦⑧⑨⑩	9,3€ Yes ①<br>0,5€ Yes ②③④⑤⑥⑦⑧⑨⑩
5	4€ Yes ①②③<br>1€ Yes ④⑤⑥⑦⑧⑨⑩	10,6€ Yes ①<br>0,5€ Yes ②③④⑤⑥⑦⑧⑨⑩
6	4€ Yes ①②③<br>1€ Yes ④⑤⑥⑦⑧⑨⑩	12,5€ Yes ①<br>0,5€ Yes ②③④⑤⑥⑦⑧⑨⑩
7	4€ Yes ①②③<br>1€ Yes ④⑤⑥⑦⑧⑨⑩	15,0€ Yes ①<br>0,5€ Yes ②③④⑤⑥⑦⑧⑨⑩
8	4€ Yes ①②③<br>1€ Yes ④⑤⑥⑦⑧⑨⑩	18,5€ Yes ①<br>0,5€ Yes ②③④⑤⑥⑦⑧⑨⑩
9	4€ Yes ①②③<br>1€ Yes ④⑤⑥⑦⑧⑨⑩	22,0€ Yes ①<br>0,5€ Yes ②③④⑤⑥⑦⑧⑨⑩
10	4€ Yes ①②③<br>1€ Yes ④⑤⑥⑦⑧⑨⑩	30,0€ Yes ①<br>0,5€ Yes ②③④⑤⑥⑦⑧⑨⑩
11	4€ Yes ①②③<br>1€ Yes ④⑤⑥⑦⑧⑨⑩	40,0€ Yes ①<br>0,5€ Yes ②③④⑤⑥⑦⑧⑨⑩
12	4€ Yes ①②③<br>1€ Yes ④⑤⑥⑦⑧⑨⑩	60,0€ Yes ①<br>0,5€ Yes ②③④⑤⑥⑦⑧⑨⑩
13	4€ Yes ①②③<br>1€ Yes ④⑤⑥⑦⑧⑨⑩	100,0€ Yes ①<br>0,5€ Yes ②③④⑤⑥⑦⑧⑨⑩
14	4€ Yes ①②③<br>1€ Yes ④⑤⑥⑦⑧⑨⑩	170,0€ Yes ①<br>0,5€ Yes ②③④⑤⑥⑦⑧⑨⑩
	I choose Plan A for Questions 1-____	I choose Plan B for Questions___ -14
Series 2	Plan A	Plan B
15	4€ Yes ①②③④⑤⑥⑦⑧⑨<br>3€ Yes ⑩	5,4€ Yes ①②③④⑤⑥⑦<br>0,5€ Yes ⑧⑨⑩
16	4€ Yes ①②③④⑤⑥⑦⑧⑨<br>3€ Yes ⑩	5,6€ Yes ①②③④⑤⑥⑦<br>0,5€ Yes ⑧⑨⑩
17	4€ Yes ①②③④⑤⑥⑦⑧⑨<br>3€ Yes ⑩	5,8€ Yes ①②③④⑤⑥⑦<br>0,5€ Yes ⑧⑨⑩
18	4€ Yes ①②③④⑤⑥⑦⑧⑨<br>3€ Yes ⑩	6,0€ Yes ①②③④⑤⑥⑦<br>0,5€ Yes ⑧⑨⑩
19	4€ Yes ①②③④⑤⑥⑦⑧⑨<br>3€ Yes ⑩	6,2€ Yes ①②③④⑤⑥⑦<br>0,5€ Yes ⑧⑨⑩
20	4€ Yes ①②③④⑤⑥⑦⑧⑨<br>3€ Yes ⑩	6,5€ Yes ①②③④⑤⑥⑦<br>0,5€ Yes ⑧⑨⑩
21	4€ Yes ①②③④⑤⑥⑦⑧⑨<br>3€ Yes ⑩	6,8€ Yes ①②③④⑤⑥⑦<br>0,5€ Yes ⑧⑨⑩
22	4€ Yes ①②③④⑤⑥⑦⑧⑨<br>3€ Yes ⑩	7,2€ Yes ①②③④⑤⑥⑦<br>0,5€ Yes ⑧⑨⑩

23	4€ Yes ①②③④⑤⑥⑦⑧⑨<br>3€ Yes ⑩	7,7€ Yes ①②③④⑤⑥⑦<br>0,5€ Yes ⑧⑨⑩
24	4€ Yes ①②③④⑤⑥⑦⑧⑨<br>3€ Yes ⑩	8,3€ Yes ①②③④⑤⑥⑦<br>0,5€ Yes ⑧⑨⑩
25	4€ Yes ①②③④⑤⑥⑦⑧⑨<br>3€ Yes ⑩	9,0€ Yes ①②③④⑤⑥⑦<br>0,5€ Yes ⑧⑨⑩
26	4€ Yes ①②③④⑤⑥⑦⑧⑨<br>3€ Yes ⑩	10,0€ Yes ①②③④⑤⑥⑦<br>0,5€ Yes ⑧⑨⑩
27	4€ Yes ①②③④⑤⑥⑦⑧⑨<br>3€ Yes ⑩	11,0€ Yes ①②③④⑤⑥⑦<br>0,5€ Yes ⑧⑨⑩
28	4€ Yes ①②③④⑤⑥⑦⑧⑨<br>3€ Yes ⑩	13,0€ Yes ①②③④⑤⑥⑦<br>0,5€ Yes ⑧⑨⑩
	I choose Plan A for Questions 15 - ____	I choose Plan B for Questions___ - 28
Series 3	Plan A	Plan B
29	Gain 2,5€ Yes ①②③④⑤<br>Loss 0,4€ Yes ⑥⑦⑧⑨⑩	Gain 3,0€ Yes ①②③④⑤<br>Loss 2,1€ Yes ⑥⑦⑧⑨⑩
30	Gain 0,4€ Yes ①②③④⑤<br>Loss 0,4€ Yes ⑥⑦⑧⑨⑩	Gain 3,0€ Yes ①②③④⑤<br>Loss 2,1€ Yes ⑥⑦⑧⑨⑩
31	Gain 0,1€ Yes ①②③④⑤<br>Loss 0,4€ Yes ⑥⑦⑧⑨⑩	Gain 3,0€ Yes ①②③④⑤<br>Loss 2,1€ Yes ⑥⑦⑧⑨⑩
32	Gain 0,1€ Yes ①②③④⑤<br>Loss 0,4€ Yes ⑥⑦⑧⑨⑩	Gain 3,0€ Yes ①②③④⑤<br>Loss 1,6€ Yes ⑥⑦⑧⑨⑩
33	Gain 0,1€ Yes ①②③④⑤<br>Loss 0,8€ Yes ⑥⑦⑧⑨⑩	Gain 3,0€ Yes ①②③④⑤<br>Loss 1,6€ Yes ⑥⑦⑧⑨⑩
34	Gain 0,1€ Yes ①②③④⑤<br>Loss 0,8€ Yes ⑥⑦⑧⑨⑩	Gain 3,0€ Yes ①②③④⑤<br>Loss 1,4€ Yes ⑥⑦⑧⑨⑩
35	Gain 0,1€ Yes ①②③④⑤<br>Loss 0,8€ Yes ⑥⑦⑧⑨⑩	Gain 3,0€ Yes ①②③④⑤<br>Loss 1,1€ Yes ⑥⑦⑧⑨⑩
	I choose Plan A for Questions 29 - ____	I choose Plan B for Questions___ - 35

Switching point (question) in Series 1 and 2, and approximations of σ (risk parameter)

**σ**	**Switching point for series 1**													
**Series 2**	1	2	3	4	5	6	7	8	9	10	11	12	13	14	Never
**1**	1.50	1.40	1.35	1.25	1.15	1.10	1.00	0.95	0.90	0.85	0.80	0.75	0.65	0.55	0.50
**2**	1.40	1.30	1.25	1.15	1.10	1.00	0.95	0.90	0.85	0.80	0.75	0.70	0.60	0.55	0.50
**3**	1.30	1.20	1.15	1.10	1.00	0.95	0.90	0.85	0.80	0.75	0.70	0.65	0.55	0.50	0.45
**4**	1.20	1.15	1.05	1.00	0.95	0.90	0.85	0.80	0.75	0.70	0.65	0.55	0.50	0.45	0.40
**5**	1.15	1.05	1.00	0.95	0.90	0.85	0.80	0.75	0.70	0.65	0.55	0.50	0.45	0.40	0.35
**6**	1.05	1.00	0.95	0.90	0.85	0.80	0.75	0.70	0.65	0.60	0.55	0.50	0.45	0.40	0.35
**7**	1.00	0.95	0.90	0.85	0.80	0.75	0.70	0.65	0.60	0.55	0.50	0.45	0.40	0.35	0.30
**8**	0.95	0.90	0.85	0.80	0.75	0.70	0.65	0.60	0.55	0.50	0.45	0.40	0.35	0.30	0.25
**9**	0.90	0.85	0.80	0.75	0.70	0.65	0.60	0.55	0.50	0.45	0.40	0.35	0.30	0.25	0.20
**10**	0.85	0.80	0.75	0.70	0.65	0.60	0.55	0.50	0.45	0.40	0.35	0.30	0.25	0.20	0.20
**11**	0.80	0.70	0.65	0.60	0.65	0.55	0.50	0.45	0.40	0.35	0.30	0.25	0.20	0.15	0.15
**12**	0.75	0.65	0.60	0.55	0.50	0.50	0.45	0.40	0.35	0.30	0.25	0.20	0.20	0.15	0.10
**13**	0.65	0.60	0.55	0.50	0.45	0.45	0.40	0.35	0.30	0.25	0.20	0.15	0.15	0.10	0.10
**14**	0.60	0.55	0.50	0.45	0.40	0.35	0.35	0.30	0.25	0.20	0.15	0.10	0.10	0.10	0.05
**Never**	0.50	0.45	0.40	0.40	0.35	0.30	0.30	0.25	0.20	0.15	0.10	0.10	0.05	0.05	0.05

NB: values less than one indicate that the individual is risk averse, values equal to one indicate the individual is risk neutral, whilst values greater than one indicate the individual is risk loving (Abducted from Tanaka *et al.*, 2010).
